# Simultaneous Correlative Scanning Electron and High-NA Fluorescence Microscopy

**DOI:** 10.1371/journal.pone.0055707

**Published:** 2013-02-08

**Authors:** Nalan Liv, A. Christiaan Zonnevylle, Angela C. Narvaez, Andries P. J. Effting, Philip W. Voorneveld, Miriam S. Lucas, James C. Hardwick, Roger A. Wepf, Pieter Kruit, Jacob P. Hoogenboom

**Affiliations:** 1 Department of Imaging Science and Technology, Faculty of Applied Sciences, Delft University of Technology, Delft, The Netherlands; 2 DELMIC BV, Delft, The Netherlands; 3 Department of Gastroenterology and Hepatology, Leiden University Medical Center, Leiden, The Netherlands; 4 Electron Microscopy ETH Zurich - EMEZ, ETH Zurich, Zurich, Switzerland; Tufts University, United States of America

## Abstract

Correlative light and electron microscopy (CLEM) is a unique method for investigating biological structure-function relations. With CLEM protein distributions visualized in fluorescence can be mapped onto the cellular ultrastructure measured with electron microscopy. Widespread application of correlative microscopy is hampered by elaborate experimental procedures related foremost to retrieving regions of interest in both modalities and/or compromises in integrated approaches. We present a novel approach to correlative microscopy, in which a high numerical aperture epi-fluorescence microscope and a scanning electron microscope illuminate the same area of a sample at the same time. This removes the need for retrieval of regions of interest leading to a drastic reduction of inspection times and the possibility for quantitative investigations of large areas and datasets with correlative microscopy. We demonstrate Simultaneous CLEM (SCLEM) analyzing cell-cell connections and membrane protrusions in whole uncoated colon adenocarcinoma cell line cells stained for actin and cortactin with AlexaFluor488. SCLEM imaging of coverglass-mounted tissue sections with both electron-dense and fluorescence staining is also shown.

## Introduction

Understanding cellular structure-function relations requires the complementary capabilities of both fluorescence and electron microscopy. Fluorescence microscopy (FM) visualizes individual proteins in color through the use of immunofluorescent or endogenous labeling [1]. Optical superresolution techniques have enabled protein localization with accuracies down to 20 nanometer [2], but intrinsic to fluorescence measurements is the fact that only the labeled components are visible. Electron microscopy (EM) on the other hand maps the cellular ultrastructure at nanometer-scale resolution. Correlative microscopy bridges the gap between optical and electron microscopy by rendering an overlay image after application of both techniques on the same area of the specimen. The possibility to map protein locations onto the cellular structure retrieved at nanometer-scale accuracy with electron microscopy, has in recent years sparked interest in correlative light and electron microscopy (CLEM) [3], [4], [5], [6], [7], [8], [9], [10], [11], [12], [13], [14], [15].

Typically, in CLEM research, inspection with FM and EM is performed on the two separate microscopes. In this way, both types of microscopy can be used at their full capabilities, including superresolution FM [13], [15]. However, CLEM procedures are arduous and require expert operation for several reasons. First, it is intrinsically difficult to retrieve a region of interest (ROI) identified with FM in EM, as the mechanisms for contrast generation in both microscopes are widely different. Thus, specialized sample holders or navigation markers are needed to facilitate ROI retrieval [11], [13], [15], [16], [17]. Second, the time involved in a CLEM experiment with transfer between both microscopes and retrieval of ROI’s typically takes several days. Third, the transfer between both microscopes makes the sample vulnerable to contamination or damage. Fourth, for re-inspection with FM after one CLEM cycle in order to identify additional ROI’s, the transfer procedure needs to be performed over again. This limits the amount of data that can be extracted in a CLEM measurement and puts strict requirements on the success rate of sample preparation and staining procedures. Last, the accuracy with which the retrieved ROIs in FM and EM images can be overlaid is limited and typically worse than the resolution of the microscopy techniques themselves. The widespread application of CLEM for examining biological structure-function relations requires simplified and routinely applicable techniques that meet the demands outlined above.

The retrieval of ROI’s can be facilitated using external markers on the sample holder that allow definition of a universal coordinate system in both FM and EM [11], [16]. The need to mount the holder in the two microscopes typically limits the accuracy in the order of micrometers. Also commercially available algorithms can be used that recognize features that are intrinsically present in both images [16]. Alternatively, fiducial markers that can be observed with both FM and EM can be used [13], [15], [17]. The definition and identification of reliable markers over large areas is not trivial and requires great care. With fiducial markers, such as fluorescent or gold nanoparticles, a ROI can be identified with high accuracy (50–100 nm) [17], but the search-and-find procedure can still be laborious and a typical research targets a single or a few ROI’s. In addition, these procedures do not target the other issues involved in CLEM.

Integrated approaches, where an optical microscope is integrated in an EM vacuum chamber, offer a practical solution to several issues. This approach was pioneered in an SEM in the early work of Wouters *et al.* [18] and recently extended to TEM by Gerritsen and co-workers [7]. In the latter microscope, called iLEM (integrated Light Electron Microscope), the sample is automatically transferred within the vacuum chamber from FM to TEM after identification of a ROI by 90° rotation of the sample stage. The integrated approach reduces CLEM process times from days to hours or less and removes the risk of sample contamination [7]. The optical microscope that can be integrated in a TEM is however necessarily low-NA and long working distance. In addition, the internal transfer from FM to EM still limits the overlay accuracy to the order of micrometers [7].

In the SEM, a high-NA optical microscope can be integrated into the vacuum chamber [18]. We have recently presented a design that gives the possibility to perform high-resolution FM inside an SEM without compromise to SEM operation. Here, we demonstrate that this integrated microscope enables a novel approach to CLEM, which relies on the possibility to apply both high-resolution light and electron microscopy *simultaneously* to the same area of a sample. While in correlative microscopy both modalities are applied sequentially, the fact that both the LM and the EM can illuminate the same area at the same time removes the need for sample transfer, ROI retrieval, and definition of markers. This procedure makes correlation unambiguous, straightforward, and fast, enabling routine application of high-resolution correlative microscopy. In addition, both optical and electron microscopy can be used at their full capabilities, extending the possibilities for quantitative FM-EM investigations of large numbers of ROI’s.

## Results

### Simultaneous CLEM

**Figure 1:**
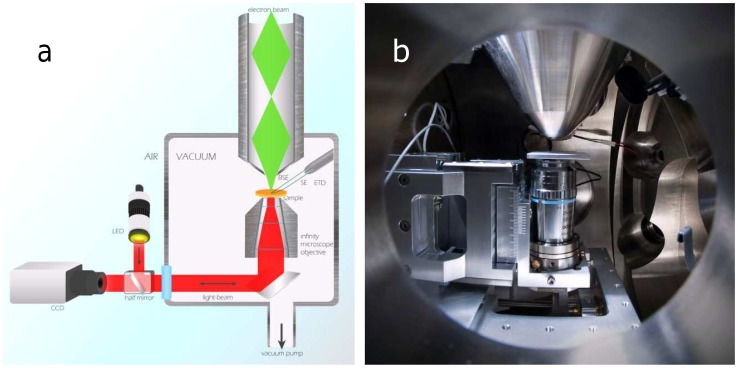
Simultaneous Correlative Light and Electron Microscopy. (a) schematic lay-out for SCLEM, BSE: backscattered electrons, SE: secondary electrons, ETD: Everhard-Thornley detector, LED: light emitting diode, CCD: charge coupled device camera. (b) inside view of the integrated microscope for SCLEM showing optical objective lens in epi-configuration underneath sample holder and electron lens.

For Simultaneous Correlative Light-Electron Microscopy (SCLEM), we use an integrated microscope where the objective lens is positioned in the vacuum chamber of a Scanning Electron Microscope (SEM), directly underneath the sample (see Figure 1a). Contrary to previous integrated solutions [7], [18], [19], the electron and optical axes are aligned parallel to each other and normal to the substrate from opposite sides. The distance between both axes is typically controlled to within 10 µm. Better axial alignment, down to 1 µm, can in principle be achieved but is not necessary as the electron axis can be shifted over the remaining distance electronically using the beam deflectors in the SEM column. Axial alignment in the micrometer range ensures that this beam shift does not introduce aberrations in the SEM image.

As can be seen in Figure 1a, the objective lens is mounted inside the SEM vacuum chamber similar to an inverted optical microscope. Illumination and detection occurs in epi-configuration. Using vacuum-compatible immersion oil, objective lenses with numerical aperture up to 1.4 can be used. Light collected by the objective lens is guided by a mirror through an optical window mounted in the door of the vacuum chamber. As is schematically illustrated in Figure 1b, components for optical illumination and detection can be arranged at will outside the vacuum chamber. In this research, light from a 470 nm LED source is collimated and sent through a dichroic mirror into the vacuum chamber where it illuminates the sample through a 100×1.4 NA objective lens immersed with a vacuum compatible immersion oil. The collected fluorescence light is directed through the dichroic mirror and focused onto a CCD camera. The SEM is operated in usual fashion with electron excitation and detection from above the sample.

Image formation in the SEM occurs through detection of either low-energy, secondary electrons (SE) or high-energy back-scattered electrons (BSE). Among other contrast mechanisms, SE imaging gives nanometer-scale detail of surface topography, while the BSE signal originates from a larger sample volume contrasting differences in atomic number or density. In our SCLEM setup, samples need to be mounted on a transparent substrate. The use of transparent conductive ITO-coated glass coverslides eliminates the need for a conductive over-coating of biological materials [8]. Cells can be cultured directly on the ITO-slides [20] and details in surface topography can be imaged without additional staining procedures. Alternatively, thin sections can be cut from a larger three-dimensional sample and mounted on the ITO-slides. In this case staining for SEM has to be performed to yield SE and/or BSE contrast. Below, we will demonstrate SCLEM for both sample types: First, uncoated, fluorescent labeled whole cells without EM staining, second thin tissue sections with both EM and FM staining.

### Cell-Cell Connections in Uncoated, Unstained Whole Cells

The formation and growth of cellular extensions and protrusions, such as filopodia, lammelipodia, and invadopodia, plays a crucial role in cell motility and cell-cell signaling. These processes involve a wide variety of proteins. The role that these proteins play in the development and maturation of cellular topography, is an area of active research. SCLEM on uncoated, whole cells may serve as a powerful technique to investigate the role of protein localization as the SEM can record a detailed map of the network of cellular protrusions.

**Figure 2:**
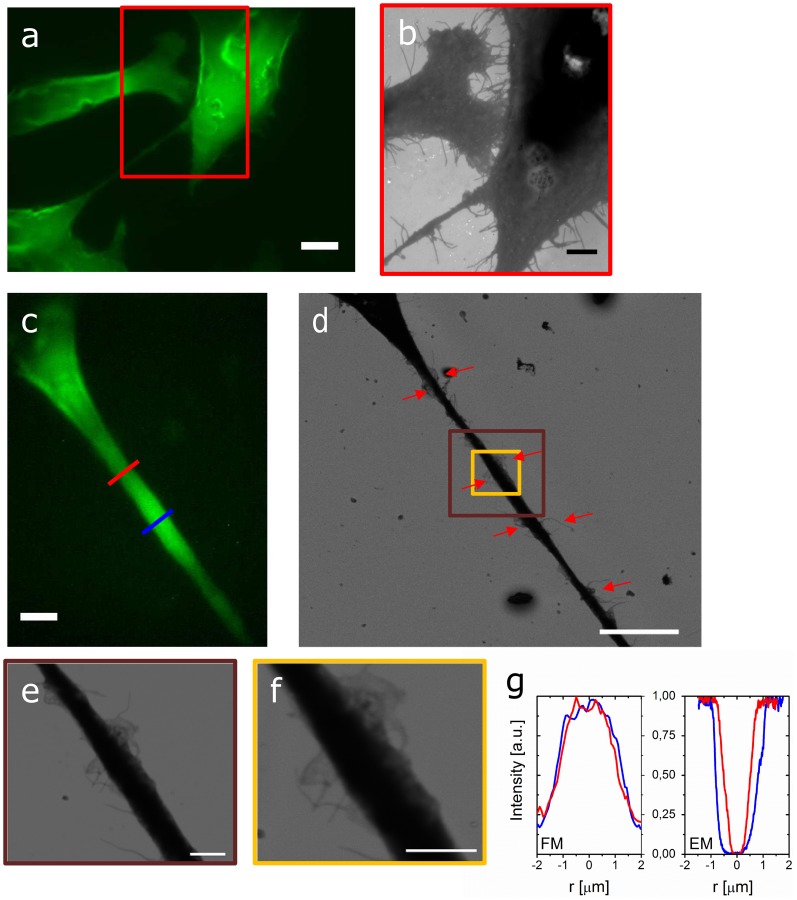
SCLEM of whole uncoated cells. (a) FM image of three adenocarcinoma cells actin labeled with Alexa488. The three cells are connected via tentacles and larger extrusions. Scalebar 5 µm. (b) SEM image of the boxed area in (a), showing detailed information on the connections between the cells. A dense network of tentacles and lamellae stretches between the upper and the right cell. Scalebar 3 µm(c) FM image of an extension connecting another two adenocarcinoma cells. Clear variations in actin concentration along the extrusion can be observed. (b) BSE image of the extrusion in (a). Red arrows mark areas with increased concentration of tentacles that occur before and after the thinner parts of the extrusion. Scale bar is 10 µm. (d, e) SE and BSE high-magnification images of the boxed areas in (b) showing a region rich in tentacles and small lamellar extrusions. Scale bars are 2 µm. (f) Fluorescence intensity profiles, normalized on the maximum, taken along the red and blue lines in (a). (g) Normalized SE intensity profile taken at the corresponding locations marked in (c).

As a first illustration of the application of SCLEM, we immuno-labeled SW480 colon adenocarcinoma cell line cells for actin with phalloidin-Alexa488. Wide-field fluorescence allows for rapid identification of labeled cells and selection of a region of interest. In Figure 2a, three nearby cells can be seen with a few actin-containing tentacles stretching out in between the cells. The cellular topography can be imaged (Figure 2b) and overlaid with the FM directly after identification of the region of interest. Note that in this image the ITO-surface appears bright due to the stronger electron scattering on indium and tin atoms [8] compared to the cellular materials. The high-magnification SEM image in Figure 2b reveals inclusions on the upper cell membrane. Importantly, the detailed network of tentacles and small lamellae connecting the cells is clearly resolved. The typical lateral size of the thin tentacles stretching between the two cells visible in Figure 2b is 60 nm.

In other cases, cell-cell connections were found to consist of larger extensions stretching several tens to hundreds of micrometers. In Figure 2c–f fluorescence and electron images of such an extension connecting two neighboring cells are shown. The fluorescence image displays variations in actin concentration and thickness of the extension. With the SEM, the lateral dimensions can be determined. Figure 2g,h shows the line profiles at the marked positions from both the fluorescence and electron images. To display and compare both curves, intensities have been normalized to their maximum. The lateral size is found to be 1 µm resp. 2 µm. In the SEM images, we also observe the outgrowth of tentacles and small lamellae from this larger extension. Interestingly, it can be seen that the outgrowth of tentacles and lamellae occurs at the positions where the filament size changes, as marked with red arrows. Figures 2e and 2f show detailed images of such a region.

### Cortactin Distribution and Cellular Topography

**Figure 3:**
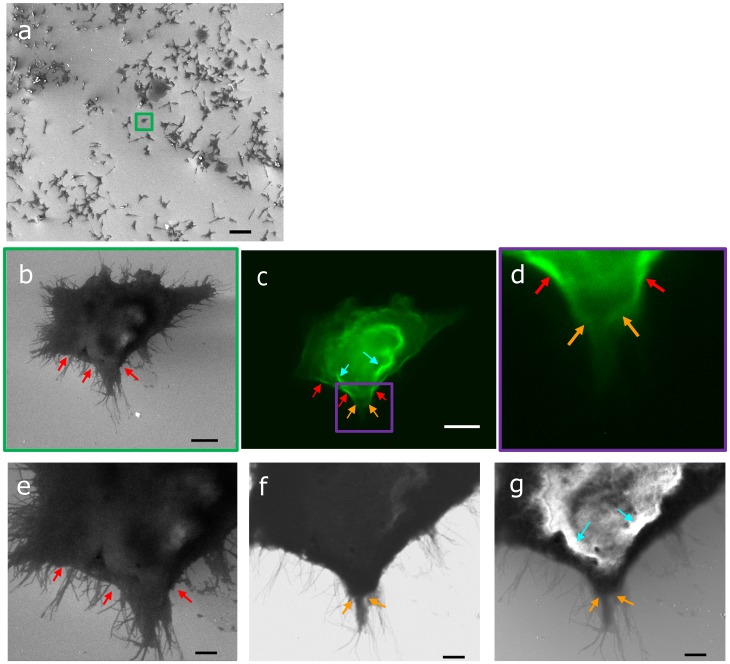
SCLEM inspection procedure with Cortactin labeled adenocancerinoma cells. (a) After mounting the sample and vacuum pumping of the SEM chamber, a low-magnification image in SEM mode is taken to inspect surface coverage and position the sample stage. (b) An isolated cell is identified and the SEM focus is fine-tuned for high-magnification imaging (c) The fluorescence image is recorded after the marked cell was selected. Based on the spatial variations in cortactin distribution and the structural overview in (b), regions of interest are identified for high-magnification imaging in SEM mode. Blue, red, and yellow arrows indicate different type of regions with a local increase in cortactin density. Corresponding areas are also marked in the SEM images. (d) SE image recorded at 20keV of the region of interest identified in (c). The cortactin-rich areas marked with red arrows are directly neighboring regions with larger extrusions and high density of tentacles (e) BSE image recorded at 5keV. (f) SE image at 5keV reveals the details in surface topography. It can be clearly seen that the blue marked cortactin-rich regions located in the cell interior correspond to an increase in cell thickness. The cortactin-rich regions marked with yellow arrows surround a larger thin lamellar outgrowth with numerous extending tentacles. Typical time involved in such a procedure (sample mounting & pump down – a,b – c – d,e,f) amounts to 20–35 minutes (4 min –5 min –5 min –5–15 min).

Next, we labeled the SW480 adenocarcinoma cells for cortactin, again with Alexa488. Cortactin is involved in rearrangement of the actin network and as such important in the formation of filopodia, lammelipodia, and invadopodia. In Figure 3b and c, we show SEM resp. FM images of an adenocarcinoma cell where cortactin is labeled with Alexa488. In Figure 3c, different regions with increased fluorescence compared to the surroundings can be observed. First, there are large areas with strong fluorescence in the cell interior, two of which are marked with blue arrows. Second, regions with increased fluorescence, several hundreds of nanometers long, stretch along the outer cell membrane. Examples of such regions are marked with red arrows. Finally, in extruding areas such as in the lower part of the cell, smaller areas with a local increase in fluorescence can be observed (marked with yellow arrows).

The same areas are marked in the SEM images in Figures 3b, and 3d–f. From the SE image in Figure 3f, it can be seen that the blue marked areas have a strong SE contrast. This indicates a large increase in cell height, as SE can only escape from a few nanometers deep. Thus the strong increase in cortactin concentration observed in the FM image, can be, at least in part, ascribed to an increase in membrane surface area. Contrarily, the variations in cortactin concentration observed at the outer membrane edge, can be directly linked to tentacles and larger extrusions of the cell membrane. In the Figures 3b and d, it can be seen that the cortactin-rich areas are adjacent to areas with more and larger outgrowth. The apex of the extrusion in the lower part of the image consists of a filopodium-like structure (Figure 3e). The location at which this structure extrudes from the membrane is again surrounded by cortactin accumulations on the cell membrane (yellow arrows). This illustrates how SCLEM can correlate protein localization to cellular extrusions and, ultimately, cell motility. The use of high-NA objective for FM enables the extraction of high-resolution fluorescence data. Moreover, as there is no specimen transfer, or re-adjustment of a ROI involved, SCLEM allows for routine inspection of a large number of cells. This will enable the extraction of quantitative CLEM data, e.g. in this case correlating position-dependent fluorescence intensity with statistics on the number, length and lateral dimensions of cellular extensions. Such investigations are currently underway.

### SCLEM offers Reduced Inspection Times and Sampling of Multiple ROI’s

As mentioned above, one of the important results of SCLEM is that there is no need for specimen transfer and re-adjustment of a ROI to combine high-NA FM data with structural data retrieved with SEM. Correlative imaging is achieved without adding fiducial markers to either the specimen support or the sample itself. This greatly simplifies the experimental workflow for CLEM and allows a user to search for a new ROI directly after inspecting another one. As a demonstration, the total time involved in a typical inspection procedure, as with the cortactin-labeled cancer cells shown in Figure 3, was measured.

Figure 3 shows a sequence of images taken in the experiment. After mounting the sample and SEM vacuum pump down, we first perform a low-magnification inspection of the sample with the SEM in order to evaluate the surface coverage of cells. This way we can determine areas on the coverglass where a substantial amount of single cells can be found (Figure 3a). After sample translation to such an area, higher magnification FM and SEM images are taken (Figure 3b–c), where the FM image serves to identify the ROIs for SEM high-resolution structural inspection. We then perform the SEM zoom-in to display the structural detail of selected parts of the cell (Figure 3d–f). This experiment, from sample mounting and vacuum pump down to full inspection, takes 20–35 minutes. The 15 minutes margin depends on the amount of high-magnification investigations that are performed with the SEM. This includes the investigation of different areas per cell, as well as various detectors (BSE and SE) and electron energies (see, e.g., Figure 3d–f). As the axial alignment between FM and SEM is fixed and the sample stage is translated, identification of and transfer to a new ROI typically only takes 5 minutes, followed by another 10–25 minutes of detailed investigations. This constitutes a drastic decrease of experiment time when compared to CLEM experiments with high-NA FM and EM on separate microscopes, where, in addition, typically only one to a few ROI’s can be sampled.

Often in the practice of FM, sample inspection is started with a low-magnification, low-NA objective lens to identify a ROI for high-resolution inspection. It is important to note that the field of view of the SEM easily extends millimeters squared and is thus much larger than that of the integrated high-NA FM. Thus, low-magnification SEM is well suited to perform a quick inspection of the sample, e.g. to analyze the surface coverage of cells (see Figure 3a). It is important to note that in Figure 3a, low-magnification SEM imaging was done before capturing the FM image in Figure 3c. Usually, fluorescence investigations are performed prior to EM to prevent accelerated bleaching during electron-beam exposure. However, similar to photo bleaching, electron-beam induced bleaching is a dose-dependent process. We observed that exposure during low magnification SEM imaging, i.e. at the multi-cellular or cellular level (cf. Figure 3a–b) does not visibly affect the fluorescence in these and other samples. This provides us with the possibility to use the large field of view of the SEM to inspect the sample for areas with a suitable coverage of cells. The sample is then translated such that this area is in the field of view of the high-NA FM. The typical inspection procedure that we use in SCLEM is depicted the sequence of images in Figure 3.

Clearly, prolonged exposure to the electron beam, such as after a high-magnification sub-cellular zoom-in, does lead to bleaching. The rate at which this occurs is dependent on electron energy, but also on the composition and thickness of the substrate and, importantly, the type of fluorophore [21]. We note that the possibility to move forth and back between FM and SEM provides a unique possibility to study electron-fluorophore interactions in detail.

### Tissue Sections with FM and EM Staining

Thin tissue sections can be investigated with SCLEM after combined FM and EM staining. Several approaches have been reported that allow for EM staining while preserving fluorescence [3], even up to the point where optical superresolution can be performed [13]. We prepared 100 nm sections of human skin stained for EM with osmium tetroxide and uranyl acetate and for FM with DiIC18. The fluorescence serves to navigate and quickly identify the corneocytes, epidermis, dermis, and other parts of the skin tissue.

**Figure 4:**
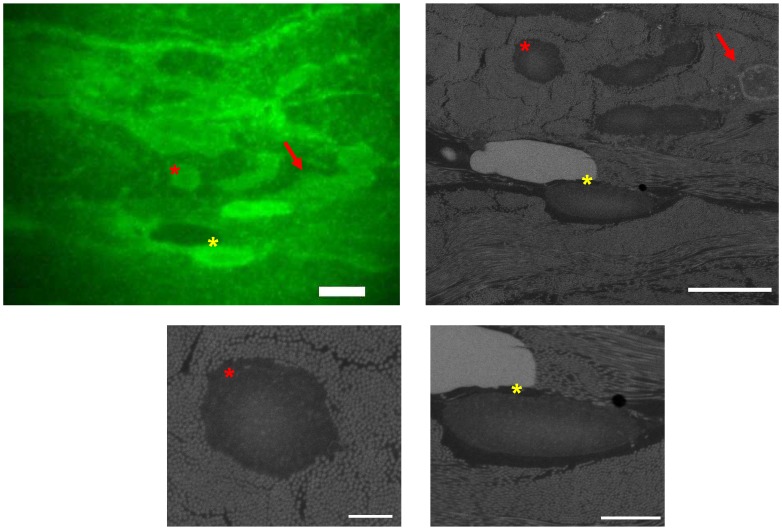
SCLEM on FM and EM stained tissue sections. (a) FM image of human skin tissue stained with DiIC18 fluorescence and uranyl acetate and osmium tetroxide for EM contrast. Scalebar 5 µm (b) BSE image of a selected region from (a), showing a cell nucleus not discernible in (a) (marked with a red arrow), and bundles of longitudinally and transversally cut collagen fibers. Scalebar is 5 µm (c, d) High-magnification images of the areas marked with (c) a red star, scalebar 1 µm, and (d) a yellow star, scalebar 2 µm.

Figure 4 shows FM and EM images of dermal tissue. In the fluorescence image structural components can be discerned based on differences in fluorescence intensity. In the middle part of the image, three lager structures, two with strong fluorescence, the other with almost no fluorescence can be identified. The corresponding SEM image clearly resolves the underlying ultrastructure in detail. Here, we note that SCLEM offers a fast procedure to identify such regions in FM and inspect the ultrastructural detail with SEM.

In the SEM image in Figure 4b various cellular constituents can be identified. A nucleus can be seen in the upper right corner. Collagen fibers running parallel to the plane of scission can be seen in the middle of the image and in the lower left corner. In other areas, such as in Figure 4c, collagen fibers, approximately 100 nm wide, are seen to run perpendicular to the plane of view. Clearly, the applied EM staining visualizes the structural detail in the tissue, while the fluorescence signal is maintained sufficiently to perform rapid tissue inspection and select regions for SEM inspection.

## Discussion

The method of SCLEM removes the need to retrieve a ROI as the alignment between SEM and FM optical axes is fixed while the sample is translated through focus. Thus, the SCLEM time for identification and inspection of a ROI is on the order of few tens of minutes, in which a user can move forth and back arbitrarily between the different SEM and FM detectors. In this way, a sample can be quickly scanned for ROI’s in either SEM or FM mode of operation and a large number of ROI’s can be investigated in a short time compared to CLEM operation on the two microscopes. In addition, issues involved in sample transfer between the microscopes, such as contamination risk, are removed from the workflow. Obviously, this also means that the sample has to be prepared to render contrast in both FM and SEM mode of operation. As we have illustrated with examples this can be done by either performing double staining, or by inspection of whole, uncoated cells with only fluorescent labeling. Alternatively, labeling with dual-contrast probes, like semiconductor quantum dots [5] or fluorescent labeled gold nanoparticles, would yield visibility in both modalities. However, the strength of EM in CLEM research is the possibility to visualize the ultrastructural detail which would then still require an additional staining step.

The development of probes and preparation protocols for correlative research has emerged in recent years. Watanabe *et al.* have demonstrated a protocol that preserves fluorescence to such an extent that superresolution fluorescence techniques like PALM and STED can be performed on EM-stained sections [13]. One of the main advantages of inspection of coverglass-mounted sections with the SEM is the possibility to analyze large arrays of sections from a 3D sample in an automated fashion. For example, in array tomography, the array of sections is inspected first in FM and then in SEM to retrieve a correlated 3D view of protein distributions and ultrastructure [22]. For 3D reconstruction, speed of operation and automation are crucial aspects. With SCLEM, the entire process of array tomography could be performed fully automated in a single pass. The development of more robust probes, more possibilities for multi-color labeling in conjunction with EM staining, or a wider palette of genetically engineered probes dedicated for CLEM applications [12], would increase the possibilities for (S)CLEM in this respect.

The surface topography of entire cells can be inspected with SEM without the need for EM staining or even conductive coating of the sample. Cells can be cultured directly on glass substrates that have a transparent, conductive ITO coating, as demonstrated by others [8], [20] and by us in this work. In principle, inspection on non-conductive glass substrates would also be possible, although inspection times would need to be short to prevent resolution loss and imaging artifacts due to charging. Alternatively, the SEM could be operated in environmental mode (ESEM) to enable electron imaging of samples that would be prone to charging. In recent years even the observation of cells under fully hydrated conditions has been shown possible using specialized sample containers with electron-transparent windows that separate the hydrated sample from the vacuum of the SEM chamber [23], [24]. A major drawback in these approaches is the destructive nature of electron-beam exposure which inhibits the observation of live-cell dynamics. Here, SCLEM would be particularly useful as a means to monitor protein dynamics prior to electron imaging. We are currently investigating such applications using a sample container with an electron transparent window opposing an optically transparent glass microscopy slide. In principle, this could also be done using sample holders for TEM with two transparent electron windows or with the atmospheric SEM developed by Nishiyama and co-workers [25].

SEM inspection of whole cells probes cellular surface structures important in cell motility and cellular signaling, such as tentacles, lammelipodia, filopodia and cell-cell connections. As illustrated in this work, SCLEM can quantitatively correlate protein distributions to densities and sizes of such surface features. As the electron beam penetrates, depending on electron energy, for several micrometers into the sample, investigation of sub-membrane structures could also be possible, albeit at progressively lower resolution. This would then require incorporation of an EM stain that generates BSE or SE contrast, like in our example of tissue sections. Still, due to scattering of the probe beam, high-resolution imaging would be limited to about 100 nm below the surface.

In the presented SCLEM set-up fluorescence microscopy is performed with a wide-field optical microscope. The low axial resolution of the wide-field microscope does not play a role in the investigation of sections or the thinner progressing or retracting parts of a cell. For samples with a thickness of a micrometer or more, the fluorescence signal may need to be optically sectioned in order to establish a correlation with the SEM signal that originates from the upper part of the sample. As most optical components, such as filters, source and detector, are placed outside the SEM vacuum chamber, illumination and detection paths can be easily adjusted or expanded without the need for vacuum-compatible components. Confocal filtering could in principle be achieved through the insertion of a pinhole. With the use of high-NA immersion objectives optical sectioning at sub-micrometer resolution should be possible. We note that also phase shaping to correct for aberrations due to refractive index differences in thick samples could be possible through the insertion of a spatial light modulator or related optics.

We equipped the fluorescence microscope with a high-NA 100× objective lens using vacuum-compatible immersion oil. The possibility to use a high-NA objective lens with coverglass-mounted samples means that total internal reflection microscopy, and superresolution techniques like PALM, could be used directly in a SCLEM experiment. For superresolution microscopy, with protein localization at a few tens of nanometer resolution, the precise positioning of proteins with respect to the ultrastructure becomes increasingly important [13], [15]. As an alternative to the ITO-coating on the coverglass, one could resort to the application of plain glass slides with a conductive coating on top of the sample as in the superresolution experiments of Watanabe *et al*. [13]. We used labeling with an Alexa-dye, but a wide range of fluorophores, including fluorescent proteins [8], can be used in conjunction with fixation protocols compatible with inspection under the SEM vacuum. A SCLEM-type set-up could thus bring the thrilling prospect of performing an optical superresolution experiment in–situ in an SEM such that at any moment the underlying cellular structure can be directly measured.

The registration between EM and FM images is a major challenge in CLEM. In the microscope we have used for our SCLEM experiments, the axial alignment between both modalities is within 10 µm. For the examples shown in this work this gives us, in combination with endogenous markers present in the sample, sufficient registration to identify and examine a ROI with both modalities. High accuracy determination could be carried out using conventional techniques, such as the use of fiducial markers [15], [17], but we anticipate that SCLEM could also offer novel approaches, such as the direct visualization of the SEM scan-region in the FM image as a result of photobleaching.

SCLEM relies on the possibility to perform both electron and optical microscopy simultaneously. We have observed that low-magnification SEM imaging at 20keV does not lead to a visible degradation of sample fluorescence. This gives us the possibility to perform wide field of view SEM inspection prior to FM investigation. Interestingly, SCLEM brings the possibility to study bleaching induced by electron-beam exposure in a quantitative and dynamic way by recording the fluorescence signal as a function of electron dose. This would not only provide a novel way of analyzing electron-induced reactions in molecules, but would also enable one to study the electron-stability of organic fluorophores and fluorescent proteins. The initial results on cathodoluminescence bleaching of organic fluorophores reported by Niitsuma *et al.* have indicated that the intramolecular electron distribution influences the electron-induced bleaching rate [21], This suggests that more irradiation resistant fluorophores could be developed, which would be particularly valuable for the development of dedicated novel probes for CLEM in general.

In conclusion, the method of SCLEM offers a fast and easy method for correlative microscopy. The same area of the sample can be illuminated by both light and electron microscope at the same time. This removes complications related to retrieval of regions of interest or the definition of fiducial markers from the correlative workflow. Inspection times are reduced to the order of minutes, there is no risk of sample contamination or damage as a result of transfer between microscopes, and a user can switch between both modalities during inspection of a region of interest. Importantly, large areas can be inspected without re-evaluation of the overlay between both images and without the need for stitching images from different areas.

We have demonstrated SCLEM with a high-NA objective lens, which allows for quantitative fluorescence microscopy in correlation to cellular ultrastructure. Equivalently, SCLEM could be performed with a large field-of-view low-NA objective lens if fluorescence labeling is solely used as a marker to track rare events suitable for EM investigation. The described implementation of SCLEM with a high-NA objective lens could be used with different optical modalities, including superresolution microscopy. We have shown SCLEM on coverglass-mounted tissue sections, as well as on whole, uncoated cells without any EM-specific staining. In the latter case, protein distributions measured in fluorescence can be correlated to the growth and size of extrusions and protrusions of the cell membrane. Thus, SCLEM could be a valuable method in the investigation of cell motility and cell-cell signaling. The ease of use and versatility of SCLEM may enable the widespread application of quantitative correlative microscopy in biology and biomedicine.

## Materials and Methods

### SCLEM

All imaging experiments were done on in-house developed optical microscope integrated in a commercial SEM (Quanta™ 200 FEG microscope (FEI, Eindhoven, The Netherlands)) as described above. SEM images were made at standard high-vacuum settings with varying acceleration voltages and different magnifications as stated in the manuscript. An Everhart-Thornley detector and a solid-state backscatter detector were used for SE and BSE detection, respectively.

Fluorescence imaging was done at room temperature using the custom made epi-fluorescence microscope which has an objective lens mounted just beneath the sample holder in the SEM chamber. The epifluorescence microscope was equipped with a 470 nm LED light source (Thorlabs M470L2-C), a CCD camera (Photometrics CoolSNAP, Tucson, Arizona, USA) and an 100X 1.4 NA objective lens (Nikon CFI Plan Apochromat VC 100x). The objective lens was tested for vacuum compatibility prior to first use. The light from the LED source passes through a collimator lens (Thorlabs LED collimator for Nikon microscopes), a planoconvex lens to focus the beam in the back-focal plane of the objective, a band-pass filter (Newport Spectra-Physics 10XM20-485), a dichroic mirror (Semrock FF506-Di03), and then through a 10 mm thick, 50 mm diameter, 425–675 nm anti-reflection coated BK7 glass window (CVI Melles Griot) into the SEzM vacuum chamber. The detection path further consists of a long-pass filter (Semrock BLP01-488R), and a standard Nikon 1X tube lens. Vacuum-compatible immersion oil was supplied by DELMIC BV (Delft, the Netherlands).

### Cell Culture

Colorectal cancer (CRC) cell line SW480 (ATCC, UK) were maintained in Dulbecco’s Modified Eagles Medium (DMEM) from Gibco Invitrogen, supplemented with penicillin (50 U/ml) and streptomycin (50 µg/ml) and 10% fetal calf serum(FCS). CRC cell line HCT116 SMAD4−/− cell line used for Cortactin labeling (obtained from Dr. B. Vogelstein - John Hopkins, Baltimore) [26] was maintained in the same way.

ITO-coated microscope slides (thickness #1, 22×22 mm with 8–12 Ωsq^−1^ or 22×40 mm with 70–100 Ω sq^−1^; SPI Supplies, West Chester, PA, U.S.A.) were washed with ethanol and water, placed in 12-well tissue culture dishes with the conductive side upwards and washed with culture medium. The cells were 2x times washed with Phosphate Buffered Saline (PBS), then trypsinized and seeded onto the ITO coated glass slides as 2 mL per well. Cells were cultured for 16–24 h at 37°C. Cells grown on ITO-coated glass at a confluency of 50%, were then washed twice with PBS containing 0.5 mM MgCl_2_, fixed for 10 minutes with a mixture of 2.5% paraformaldehyde and 1.25% glutaraldehyde in PBS, pH 7.4. Samples were washed 3 times with PBS after fixation.

### Fluorescent Labeling

Staining actin with phalloidin (Alexa Fluor 488 phalloidin; Invitrogen, Carlsbad, CA) was performed according to manufacturer's instructions. 5 µL 6.6 µM stock solution was diluted into 200 µL PBS for each coverslip and 1% bovine serum albumin (BSA) was added to the staining solution to reduce nonspecific background staining. The staining was carried on for 30 minutes at room temperature and afterwards samples were washed 3 times with PBS.

For immunolabeling of cortactin, cells were pre-incubated with PBS with 1% BSA and 0.1% Triton for 10 min, then incubated with the primary antibody in PBS/BSA/Triton for 1hr at dilution 1∶200 at room temperature. Cells were washed 3 times with PBS containing 1% BSA and 0,1% Triton. The cells were then incubated with the secondary antibody dissolved 1∶200 in PBS/1% BSA/0,1% Triton for 30 minutes at room temperature and then washed again 3 times with PBS containing 1% BSA and 0,1% Triton. The primary antibody used was Anti-Cortactin (p80/85) (mouse), clone 4F11(Millipore, MA, USA) and the secondary antibody was Alexa fluor 488 goat anti-mouse IgG (H+L) (Invitrogen,NY, USA). After labeling the samples were 3 times washed with dH_2_O and left in dH_2_O at 4°C overnight to remove any remaining salt residue from the sample.

The samples were air dried. Before imaging, conductive carbon tape was used to connect the upper, ITO-coated side of the microscope slides holding the sample to the sample holder of the SCLEM platform.

### Tissue Sections

One of the authors (R. A. W.) took samples of human skin from his own arm using standard 2 mm biopsy punches. The tissue samples were high-pressure frozen, freeze-substituted in acetone, and embedded in HM20. During freeze-substitution it was stained with osmium tetroxide, uranyl acetate, and DiIC18. Freeze-substitution was performed as follows: 27 hours at −90°C, temperature rise to −60°C at 10°C/hour, 6 hours at −60°C, temperature rise to −40°C at 10°C/hour, 5 hours at −40°C. Then the stains were washed out and infiltration was started with HM20 (30% and 70% in ethanol, and then 100% overnight). Polymerization was done with UV-light at −40°C for 3 days. 100nm sections were cut and transferred to ITO-coated thickness #1 glass cover slides. Before imaging, they were connected to the sample holder of the SCLEM platform with conductive carbon tape.
